# “Draw the sea…”: Children’s representations of ocean connectivity in Fiji and New Caledonia

**DOI:** 10.1007/s13280-022-01777-1

**Published:** 2022-09-23

**Authors:** Elodie Fache, Susanna Piovano, Alisi Soderberg, Malakai Tuiono, Léa Riera, Gilbert David, Matthias Kowasch, Simonne Pauwels, Annette Breckwoldt, Stéphanie M. Carrière, Catherine Sabinot

**Affiliations:** 1 SENS, IRD, CIRAD, Univ Paul Valery Montpellier 3, Univ Montpellier, Montpellier, France; 2 School of Agriculture, Geography, Environment, Ocean and Natural Sciences (SAGEONS), The University of the South Pacific, Laucala Bay Road, Private Mail Bag, Suva, Fiji; 3 Leibniz Centre for Tropical Marine Research (ZMT), Fahrenheitstraße 6, 28359 Bremen, Germany; 4 UMR ESPACE-DEV, Univ Montpellier, IRD, Univ Antilles, Univ Avignon-Pays de Vaucluse, Univ Guyane, Univ La Réunion, Univ Nouvelle-Calédonie, Montpellier, France; 5 Institute of Secondary Teacher Education, University College of Teacher Education Styria, Hasnerplatz 12, 8010 Graz, Austria; 6 Inland Norway University of Applied Sciences, Hamar, Norway; 7 UMR ESPACE-DEV, Univ Montpellier, IRD, Univ Antilles, Univ Avignon-Pays de Vaucluse, Univ Guyane, Univ La Réunion, Univ Nouvelle-Calédonie, Montpellier, France; 8 Humboldt-Universität zu Berlin, Berlin, Germany; 9 Aix-Marseille Université, CNRS, EHESS, CREDO (UMR 7308), Labex Corail, Marseille, France; 10 Centre IRD Anse Vata, BPA5, 98848 Nouméa Cedex, Nouvelle-Calédonie

**Keywords:** Children, Connectivity, Fisheries, Local ecological knowledge, Oceania, Pacific Ocean

## Abstract

In the South Pacific region, marine territories and resources play a crucial role for local communities. Children engage with these territories and resources from an early age onwards. As the next ocean stewards, they are a stakeholder group whose understandings of ocean connectivity and fisheries should be given serious consideration in decision-making processes towards the sustainable use and management of coastal seas. This paper analyses 290 children’s drawings from Fiji and New Caledonia, created in 2019 in spontaneous response to the instruction: “Draw the sea and what you and others do in the sea”. Exploring the webs of connections with and within the sea revealed by these children’s drawings and their own interpretations leads us to discuss children’s representations of the sea: (1) beyond a land-sea compartmentation, (2) as a locus of both exploitation and conservation of marine life, and (3) as a ‘place-full’ space connecting human and more-than-human realms.

**Supplementary Information:**

The online version contains supplementary material available at 10.1007/s13280-022-01777-1.

## Introduction

In the context of the unequalled rush for space and resources it has faced in recent decades (Fache et al. 2021), the Pacific Ocean has been the focus of three competing broad views: first, a ‘force field’ where “no one state has sovereignty” and both movement and trade are free; second, a space of capitalist accumulation, management and conservation; third, a ‘place-full’ space, “densely connected and networked, and inseparable from history, society, political and cultural identities” (Allen et al. 2018, p. 4). New pan-Pacific movements make a case for this third view, while resisting the two others, as well as the direct and indirect impacts of climate change (Teaiwa 2018). These movements are built on and linked to Epeli Hau’ofa’s claim that Oceanians are the “custodians of the ocean”, protecting it “for the general welfare of all living things” and “for the general good” (Hau’ofa 2000, pp. 33, 40), which poses an equivalence between Oceanian and Oceanic identity (Bambridge et al. 2021). This claim and respective enactments of rights over, responsibilities towards, and stewardship of the Pacific Ocean, are critical to meet global conservation goals (*ibid.*), such as the United Nations’ Sustainable Development Goal (SDG) 14, ‘Life below water’, which aims to conserve and sustainably use the oceans, seas and marine resources, while being related to all other SDGs (Singh et al. 2018), particularly to SDG 15 (‘Life on land’), SDG 13 (‘Climate action’), and SDG 12 (‘Responsible consumption and production’). Such rights, responsibilities, and stewardship are based on the reciprocal relationships between Oceanian peoples, “the sea that surrounds [their] island communities”, and “all living things” therein (Hau’ofa 2000, pp. 37, 40; Bambridge et al. 2021). As both representatives and potential providers of future generations, children are key players in maintaining these reciprocal relationships in both the near and more distant future, in a rapidly changing environment and climate.

In this paper, we ask how children in Fiji and New Caledonia experience and understand relationships between the sea, people, and marine life. More specifically, we examine to what extent children frame these relationships along a land-sea continuum, as embedded in Fiji in the *iTaukei* (Indigenous Fijian) concept of *vanua*, and in New Caledonia in *Kanak* terms such as *hnyei* (in *iaai* language) or *manaha* (in *fagauvea* language). The first refers to a holistic and complex set of social-ecological and socio-cosmic relationships (Tuwere 2002; Nabobo-Baba 2006), while the latter refer to a land-sea country or territory traversed by networks of alliances, customary paths connecting both individuals and clans, and trails used by both the living and the dead (Dégremont and Sabinot 2020). We also explore to what extent children articulate these relationships and networks with fisheries, including their own fishing practices (usually starting at a very young age; e.g., Kronen 2004; Sabinot et al. 2022), as well as with other uses of marine places and resources. The sustainability of both coastal and offshore fisheries is indeed vital to the future of Pacific Island countries and territories, but is increasingly threatened by overexploitation, marine pollution, habitat destruction, and climate change (e.g., Veitayaki and Ledua 2016). Finally, in the regional context of a worrying loss of Indigenous Fishing Knowledge (IFK) that “has been fundamental to environmental, cultural and livelihood sustainability of Pacific peoples for millennia” (Kitolelei et al. 2021, p. 1), it is critical to investigate children’s representations of interactions with and among marine life. We address these questions based on a corpus of children’s drawings from Fiji and New Caledonia, as well as the drawers’ own descriptions of their artwork.

Children’s drawings are a task-based method that facilitates children’s “ability to communicate their view of the world” while allowing them some “control over their form of expression” (Punch 2002, pp. 337, 331). In constructivist learning approaches, this method allows educators and teachers to consider and build on children’s perspectives and experiences (Schule and Felzmann 2013). In ethnoecological studies, this method has been used to explore children’s ecological knowledge (e.g., Carrière et al. 2017), from their general views of ‘nature’ (Pagezy et al. 2010) to their relationships with specific features or beings of their surroundings (such as an underwater volcano in Calandra 2013, or the animals that symbolize the forest in Dounias 2007). In Oceania, scholars have also analyzed children’s drawings on topics as varied as their favorite things (Marshall and Aitken 2006), their ideal learning environment (Bland 2012), a scientist or a group of scientists (Jane et al. 2007), the human figure (Martlew and Connolly 1996), or culture (Soukup 2011). In Fiji and New Caledonia, children’s drawings have also proved fruitful in research focused on very specific issues. In Fiji, drawing has been part of a set of tools aiming to investigate “the way children come to make relations in space isomorphic with hierarchical relations” (Toren 1990, p. 18). In New Caledonia, children’s drawings have allowed to assess the impacts of an environmental awareness campaign on children’s representations of coral reefs (Chabanet et al. 2018).

These contributions show that children’s drawings are a relevant tool for exploring the insights of the youngest part of the Pacific population on connections between an “Oceanian sovereignty” (Bambridge et al. 2021) and marine sustainability; an under-researched topic and knowledge gap this paper addresses. Children—and more generally the youth (e.g., Strand et al. 2022)—do not have a stake in decision-making processes, both globally and locally, yet their views of sustainable futures should be considered. Article 12 of the United Nations Convention on the Rights of the Child (UNCRC)^1^ states that children have the human right to express their views in all matters affecting them, and to have these views taken seriously (rather than dismissed on the grounds of age). The next step is the recognition that children have the right to participate in designing alternatives for the future, including in relation to environmental and sustainability issues (Hart 1997). The concept of sustainable development itself, with its focus on the needs of future generations, embraces aspects of intergenerational justice (Brundtland 1987). Intergenerational justice is also claimed by young climate activists, who (will) have to live with the impacts of global climate and environmental changes, and therefore raise their voices for justice (Kowasch et al. 2021). The notions of justice (Kopnina 2014) and particularly environmental justice (Schlosberg 2004; Svarstad and Benjaminsen 2020), as well as SDG 10 (‘Reduced inequalities’), are clearly concerned with equalizing relations between those who have power and those who do not, including children. The latter are increasingly experiencing eco-anxiety, i.e., a chronic fear of environmental doom, often coupled with a fear of the inability of governments and leaders to take urgent action to address the situation, which puts their future at stake (Glausiusz 2022). Against this backdrop, drawing is a research tool allowing children to express their perceptions on sustainable futures, including marine sustainability, which could potentially become a stepping stone to participation in decisions on the matter, at least at the local level.

In this paper, after a brief introduction of our study sites and methods, the interdisciplinary presentation and discussion of our results reveal that, through the mediation of their drawings, children from Fiji and New Caledonia showed us that they conceive the sea: (1) beyond a land-sea compartmentation, (2) as a locus of both exploitation and conservation of marine life, and (3) as a ‘place-full’ space connecting human and more-than-human realms.

## Materials and methods

### Study sites

**Fig. 1:**
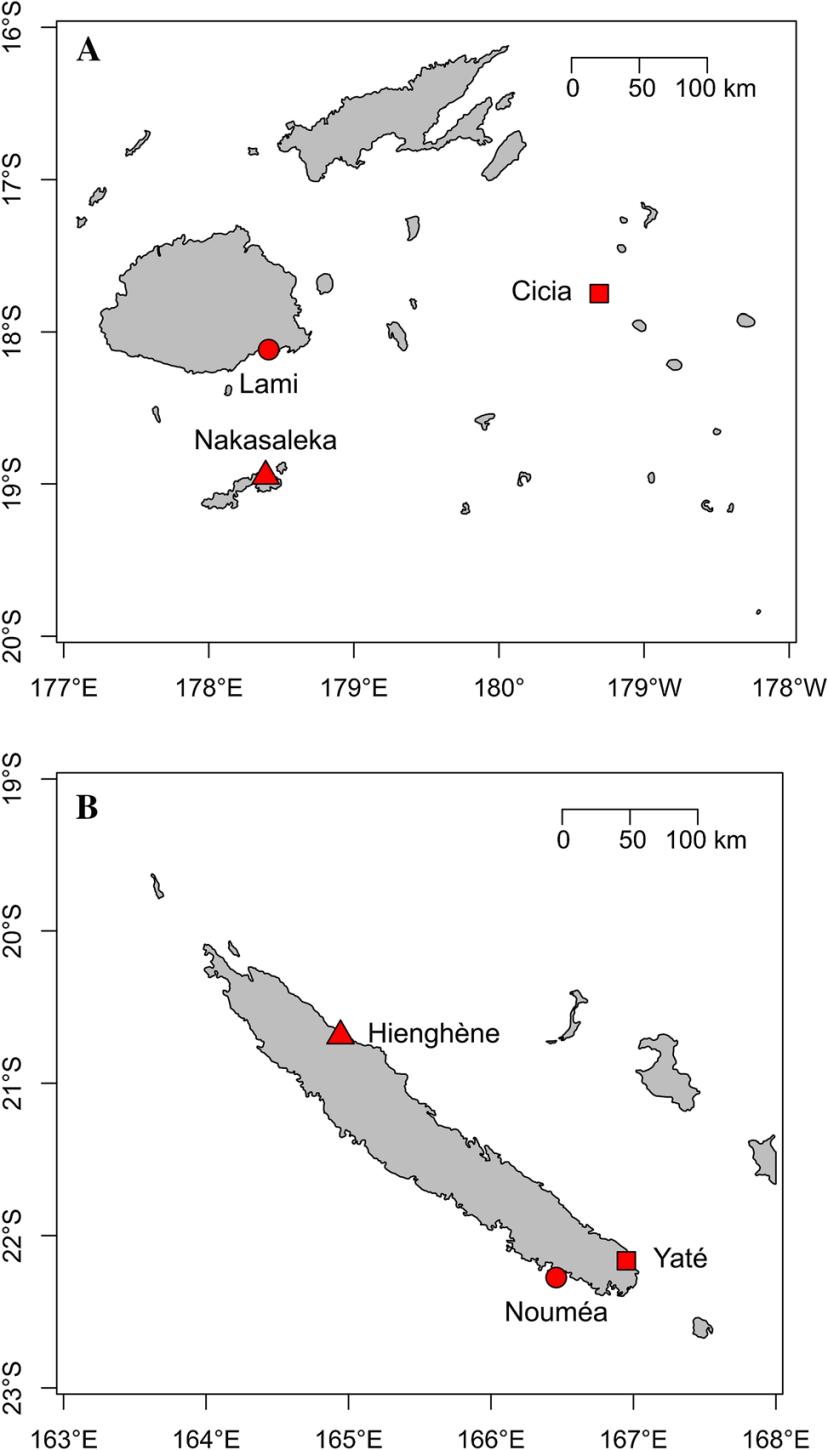
Maps of Fiji (**A**) and New Caledonia (**B**) highlighting our study sites. Circle = urban site; square = rural site; triangle = rural-MPA site

In 2019, our interdisciplinary team (anthropology, ethnoecology, geography, marine science) organized drawing workshops in schools located in three different areas in Fiji and New Caledonia: (1) an urban site; (2) a rural site; and (3) a second rural site, adjacent to a formal marine protected area (MPA) (hereafter, ‘rural-MPA site’). For Fiji, these areas were, respectively: (1) Lami, in the outskirts of Suva, the country’s capital; (2) Cicia Island in the Lau group; and (3) Nakasaleka district on Kadavu Island, adjacent to the Naiqoro Passage Spawning Aggregation Marine Reserve (hereafter, ‘Naiqoro Reserve’) (Fig. 1A). For New Caledonia, these areas were, respectively: (1) Nouméa, the capital city; (2) Yaté, about 50 km away from Nouméa; and (3) Hienghène, more than 350 km away from Nouméa, located within the *Zone Côtière Nord-Est* (ZCNE; North-Eastern Coastal Zone) (Fig. 1B).

In both Fiji and New Caledonia, an increasingly large part of the population lives in the capital city or its outskirts (Fiji Bureau of Statistics 2018; Insee 2020), hence the inclusion of urban sites in the study. Fiji’s and New Caledonia’s coral reef ecosystems host substantial marine and coastal biodiversity, are essential to local livelihoods, but are increasingly threatened by rapid social-ecological changes, as on Cicia Island and in Yaté. We also selected rural-MPA sites as we expected them to influence children’s experiences and representations of the marine world and fishing. In Fiji, the Naiqoro Reserve (4830 km^2^) was created and officially launched in 2018 by the Fijian Government, with the purpose of “conserving, protecting and maintaining the biodiversity and productivity of the species of fish, sharks, rays, cetaceans, sea turtles and all marine organisms including coral and holothurian species within the demarcated area”^2^. Within it, any fishing and gleaning activity, and more generally the collection of any marine organism including coral, is prohibited. In New Caledonia, the ZNCE (3714 km^2^) is one of the six marine clusters highlighted by UNESCO as part of the inscription of the New Caledonian lagoons on the World Heritage List in 2008^3^. The ZNCE contains different ecosystems (forests, catchment areas, mangroves, seagrass meadows, barrier and fringing reefs), some of which are under (various forms of) customary protection. Overall, it presents a lower and more diverse level of protection than the Naiqoro Reserve.

### Combining drawings and interviews

Prior to the drawing workshops, we obtained permit from government institutions and support from all school principals and teachers involved, as well as consent from the parents or guardians of all participants. A total of 290 children participated, 153 in Fiji and 137 in New Caledonia (Table S1), with an equal participation of boys and girls intended in all sites. Our target group encompassed children between 9 and 15 years old; however, in Fiji most of the participants (85%) were aged 10 to 13, while in New Caledonia most of the participants (96%) were aged 9 to 12.

Our methodology and additional epistemological considerations are presented in detail in another publication (Fache et al. 2022). We gave the following drawing instruction to all children: “Draw the sea and what you and others do in the sea”^4^. After completion of the drawings, individual semi-structured interviews were conducted to allow all children to comment on what they had just drawn, and to briefly describe their family context. These interviews lasted up to about ten minutes and were not recorded: notes were taken during each interview, often with the support of a quickly sketched schematic representation of the various elements of the respective drawing, and completed immediately afterwards. Semi-structured interviews were also conducted with principals and teachers in order to get insights into the features of each school, the profiles of its students, and the curriculum and recent activities related to marine environments.

For the processing and analysis of the dataset, we created an Excel spreadsheet coding the data related to the content of each drawing (as described by its drawer). This included the identity of the persons drawn, types of sea-related activities, fishing techniques, targeted or caught species, as well as the presence or absence of terrestrial elements, an underwater view, various forms of marine life, and marine management issues or measures. This allowed us to inductively identify three main themes in the drawings, related to connections (1) between the land and the sea, (2) between land-sea territories and fishing, including concerns about the sustainability of fisheries, and (3) between people, fish, and habitats.

### An interdisciplinary approach

Overall, this methodology has been developed to facilitate the involvement of a team of researchers (from early career to senior), from different countries and with various scientific backgrounds and experiences, either at a specific stage (e.g., designing the methodology, conducting a drawing workshop, analyzing the data) or throughout the process, with the aim to work together on common questions and to obtain shared results, as well as to jointly highlight children’s IFK and views of relationships between the sea, people, and marine life. This cooperation has allowed us to transgress disciplinary boundaries and draw on the complementarities of our multi-faceted approaches to children’s drawings. Its outcomes include the building of several bridges, notably between qualitative and quantitative analysis, between insights on fishers-fish relations and insights on interactions between marine megafauna and their habitats, and between sociocultural and ecological dimensions of ocean connectivity.

## Results and discussion

The results reflect the composition and content of the drawings, as well as the description and interpretation the children shared with us in the interviews. Some of these results are quantitative, others purposely qualitative. The interdisciplinary discussion is introduced progressively throughout the presentation of the results.

### Beyond a land-sea compartmentation

Although we asked the participants to “draw the sea…”, 76% of all children’s drawings represented both marine and terrestrial environments (Fig. S1), with varying percentages of drawings representing only marine environments (Table 1). Children who represented the land in their drawings did so by including different types of terrestrial elements: high islands, islets, or sandy beaches; vegetation (mangroves, coconut and other trees, plantations); houses and other buildings (e.g., a church, a lifeguard station, a restaurant); and/or coastal infrastructures (such as a seawall, a pontoon, a jetty, a road). These children’s representations of the sea as inextricably tied to the land—or, in other words, of land-sea territories—reflect the local worldviews we encountered in our ethnographic work with adults as well as in the scientific literature on the *iTaukei* concept of *vanua* (Tuwere 2002; Nabobo-Baba 2006) and on how *Kanak* people dwell and engage in the environment (Dégremont and Sabinot 2020).

**Table 1:** For each study site, percentage of drawings representing only marine environments (NC = New Caledonia)

	Fiji, Urban (Lami)	Fiji, Rural (Cicia)	Fiji, Rural with MPA (Kadavu)	NC, Urban (Nouméa)	NC, Rural (Yaté)	NC, Rural with MPA (Hienghène)
Percentage of ‘sea only’ drawings	22%	9%	46%	16%	32%	16%

Whether or not their drawings explicitly referred to the land, the children depicted some elements that inherently reflected different types and aspects of land-sea interactions such as boats, sea turtles, or land-originated marine pollution. For instance, 33 out of 50 drawings from the Fijian rural-MPA site included small-motorized boats (e.g., Fig. S2). Sea turtles were present in 91 drawings, i.e., in 31% of all drawings (e.g., Figs. S2 and S3). Among the 23 children who expressed—in their drawings and/or interviews—concerns about marine pollution and its impacts on marine life, three girls from the Fijian rural-MPA site highlighted waste management issues in island settings, resulting in some villagers dumping rubbish on the coastline (e.g., Fig. S4). While some boats depicted by the children are those used by local fishers to reach their fishing grounds from the village, sometimes also to bring their fish to the capital city to sell them, others are industrial fishing vessels, patrol ships, ferries, or even cruise ships. All these boats represent enactments of intra- and inter-island dynamics of resource harvesting, trade, surveillance, tourism, but also of sociality. Regarding sea turtles, they move into the open ocean as hatchlings, transition to shallow coastal habitats as juveniles and, as nesting females, return to the beach where they were born (Lutz and Musick 1996; Piovano et al. 2019), thus reflecting seascape connectivity between beaches and the open ocean, and the importance of seagrass meadows for foraging in coastal waters (Papale et al. 2020; Piovano et al. 2020). References to waste dumping and its impacts on marine life illustrate that land-based sources of pollution are a critical threat to coral reef ecosystems (Carlson et al. 2019; Dutra et al. 2021), and call for a better incorporation of land-sea processes into efforts to ensure the health of reefs and the sustainability of reef fisheries, for instance through ridge-to-reef approaches (either formal or informal; Fache and Pauwels 2022).

During the interviews, the children did not systematically specify the place they depicted, which was sometimes imaginary (Fig. S5). In cases where children identified the place (184 drawings, i.e., 63% of all drawings), it was often a place located near their house or school (125 drawings), but it was sometimes a place located in another part of the country (46 drawings), especially the home island or village of their parents and grandparents, where they had already stayed for weekends, holidays or longer periods. This suggests that children grasp land-sea connections through their daily experience and/or through their kin-based networks and interactions. The very choice to draw these specific places can be interpreted as an evidence of attachment to them (Muktiwibowo and Laskara 2018), as “their meaningful environments” (Scannell and Gifford 2010, p. 1). Places located near the children’s house or school are those they frequent most and with which they are most familiar; they might offer them positive experiences (e.g., as a space for entertainment) as well as a sense of well-being and security (Muktiwibowo and Laskara 2018). Children’s depiction of the home island or village of their parents and grandparents can reflect a “desire to remain close” to these places, which afford and symbolize family and social bonds, and thus a “proximity-maintaining behaviour” (Scannell and Gifford 2010, p. 4). These two types of person-place bonds seem to provide children with a sense of both physical and social rootness and belongingness (Scannell and Gifford 2010). Still, diverse variables might affect the children’s level or quality of attachment to these important places, such as environmental issues or electronic media (Scannell and Gifford 2010; Muktiwibowo and Laskara 2018).

### The sea as a locus of both exploitation and conservation of marine life

The children’s drawings reflected various human uses of land-sea territories, such as fishing, swimming and bathing, water sports (e.g., recreational diving, sailing, surfing), beach games (e.g., volleyball, soccer, playing with sand), picnicking and relaxing, or transport of people or goods by boat. While we intentionally omitted any direct reference to fisheries in the drawing instruction, fishing is a recurrent theme in the children’s drawings. The participants depicted fishing activities in 70% of the drawings made in Fiji, and in 29% of the drawings made in New Caledonia (Fig. S6), with part of them combining fishing with other sea-related human activities. This shows that, in both Fiji and New Caledonia, children are aware that fishing is a cornerstone of local ways of living from and with the sea.

Based on the children’s descriptions of their artwork in interviews, we were able to define four categories of fishers represented in the drawings that include fishing activities (or ‘fishing drawings’): (1) the children themselves, fishing alone; (2) the children themselves, accompanied by other people; (3) the children’s family members or friends (with the children themselves not represented in the scene); or (4) fishers unrelated to the children or unspecified. In Fiji, fishers belonged to this last category in about 40% of the fishing drawings from the urban site, whereas about 40% of the fishing drawings from the rural and rural-MPA sites featured the children themselves accompanied by other people, mainly their parents, brothers and sisters, or friends. In New Caledonia, in about 60% of the fishing drawings from the urban site and 75% from the rural-MPA site, the fishers were unrelated to the children or unspecified, while about 45% of the fishing drawings from the rural site featured the children themselves accompanied by other people, mainly their parents or grandparents. The study thus confirms that at least some children engage in fishing from an early age onwards, often accompanied by relatives (from the same or previous generations) or friends. Yet, this engagement is more prevalent among children living in a coastal rural village (such as on Cicia and Kadavu Islands in Fiji and in Yaté in New Caledonia) than among those living in a coastal urban area (Lami and Nouméa) or coming from an inland rural area (which is the case for a number of children attending school in Hienghène).

**Fig. 2:**
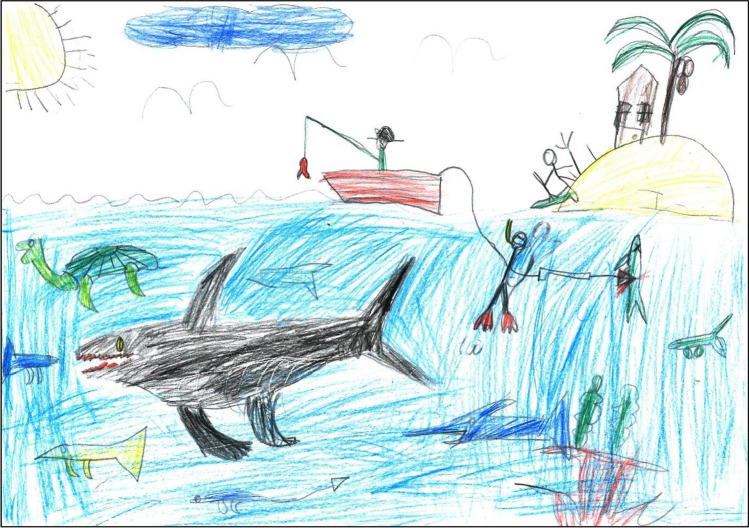
*“La mer dorée”*—“The golden sea” (drawing made by an 11-year-old boy in New Caledonia’s urban site in November 2019). In the centre of this picture, a character is line-fishing from a watercraft floating on the surface of the sea. Below on the right, another character is fishing underwater, using a speargun. Above it, still further right, a third person is fishing from the shore of a small island, using a hand-spear

In both Fiji and New Caledonia, most of the children who included small-scale fisheries in their drawing represented only one or two fishing techniques. The most commonly drawn techniques were: line-fishing from a watercraft (76 drawings); line-fishing from the shore (42 drawings); and using a speargun while free-diving (40 drawings). In the seven drawings representing more than two (i.e., 3 to 5) fishing techniques, these were distributed between different spaces and thus between different parts of the respective drawing, as illustrated by Fig. 2. More generally, in the children’s drawings, fishing techniques differ according to places and habitats, or vice versa, as well as to other parameters, including target species, availability of a watercraft, weather, or tide. These techniques also reflect the adaptation of coastal fishers in Fiji and New Caledonia to the high interspecific diversity but low intraspecific abundance of coral reef fish (David 2008).

Whereas girls drew fishing as frequently as boys in Fiji, New Caledonian girls drew fishing less often than boys: 8% vs. 32% in the urban site; 17% vs. 45% in the rural-MPA site; 24% vs. 45% in the rural site (Fig. S7). In fishing drawings where children did not represent themselves fishing alone (125 drawings), girls drew—apart from themselves—in almost equal proportion: girls or adult women fishing (12 drawings), boys or adult men fishing (16 drawings), both female and male fishers (14 drawings), and characters whose gender was not specified (13 drawings), whereas boys drew—apart from themselves—mainly boys or adult men fishing (52 out of 70 drawings) (Fig. S8). This near-absence of female fishers in the boys’ drawings can be related to the observation that, while women play crucial roles in small-scale fisheries, their contributions “continue to be *invisible*, *ignored* and *unrecognized*” (Thomas et al. 2021, p. 7). Among the 13 (out of 125) drawings that represented only girls or adult women fishing (other than the drawers themselves), six showed the latter involved in line-fishing from a watercraft, six line-fishing from the shore, and one gleaning shellfish. Among the 68 (out of 125) drawings that represented only boys or adult men fishing (other than the drawers themselves), 57 involved only one fishing technique, notably: line-fishing from a watercraft (in 22 drawings), using a speargun while free-diving (in 18 drawings), and line-fishing from the shore (in seven drawings). Among the 16 (out of 125) drawings that represented both female and male fishers (other than the drawers themselves), five reflected a clear gender differentiation in fishing locations and techniques (Fig. S9). For example, in those representing both female and male line-fishers (e.g., Fig. S9A and S9C), the girls or adult women fish from the shore, while the boys or adult men do so from a watercraft. These data reflect that both female and male fishers are involved in line-fishing from the shore or from a watercraft, but male fishers tend to fish further away from home and in deeper waters, and only boys and adult men fish underwater using a speargun. The drawings thus suggest that children are conscious of gender norms regarding the use of fishing gear and techniques. However, the study does not allow for an assessment of potential discrepancies between the normative and actual gendered division of labor in small-scale fisheries, in particular among children (e.g., does this division become more pronounced with age?).

**Fig. 3:**
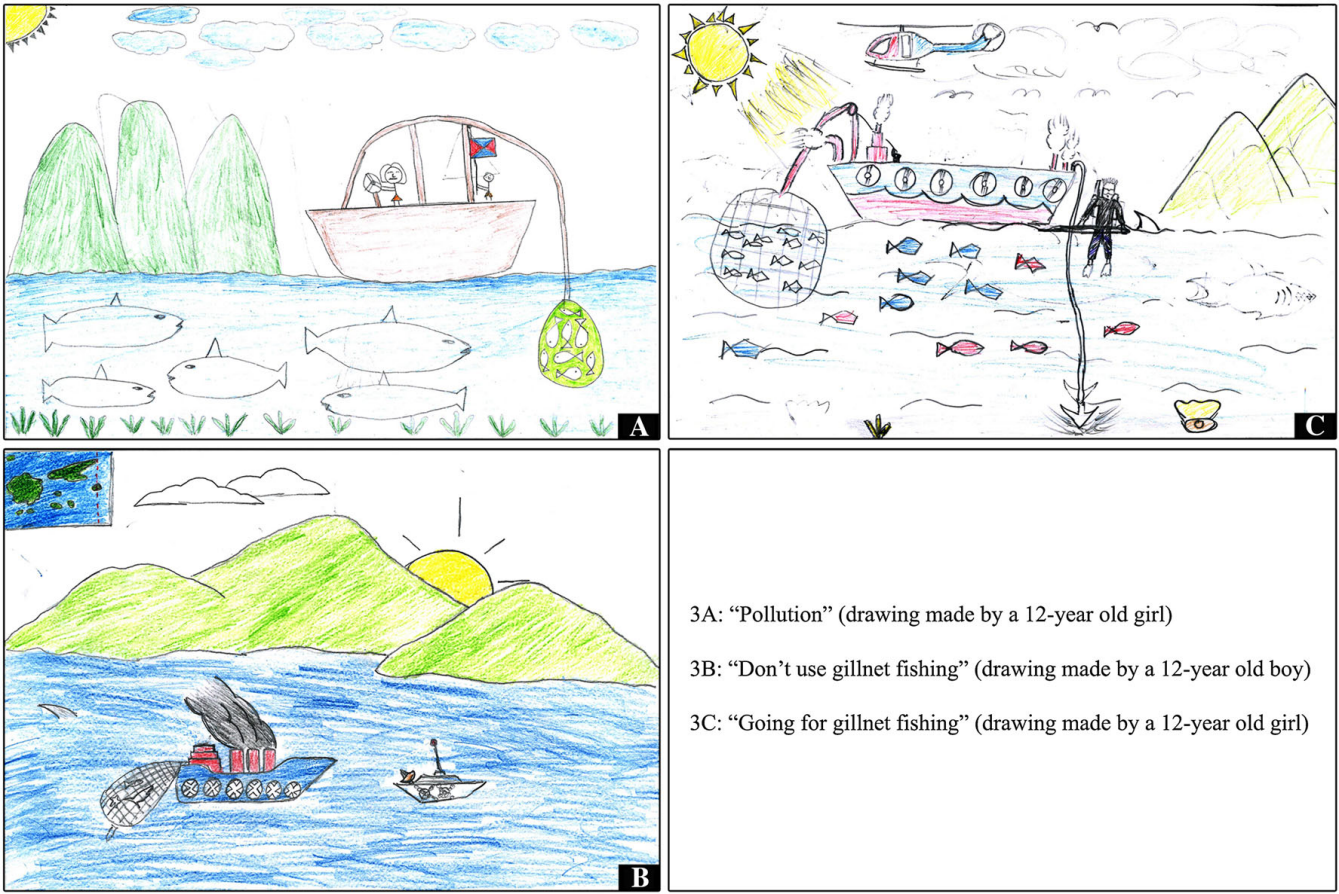
Three representations of “gillnet fishing” made in Fiji’s urban site in September 2019

**Fig. 4:**
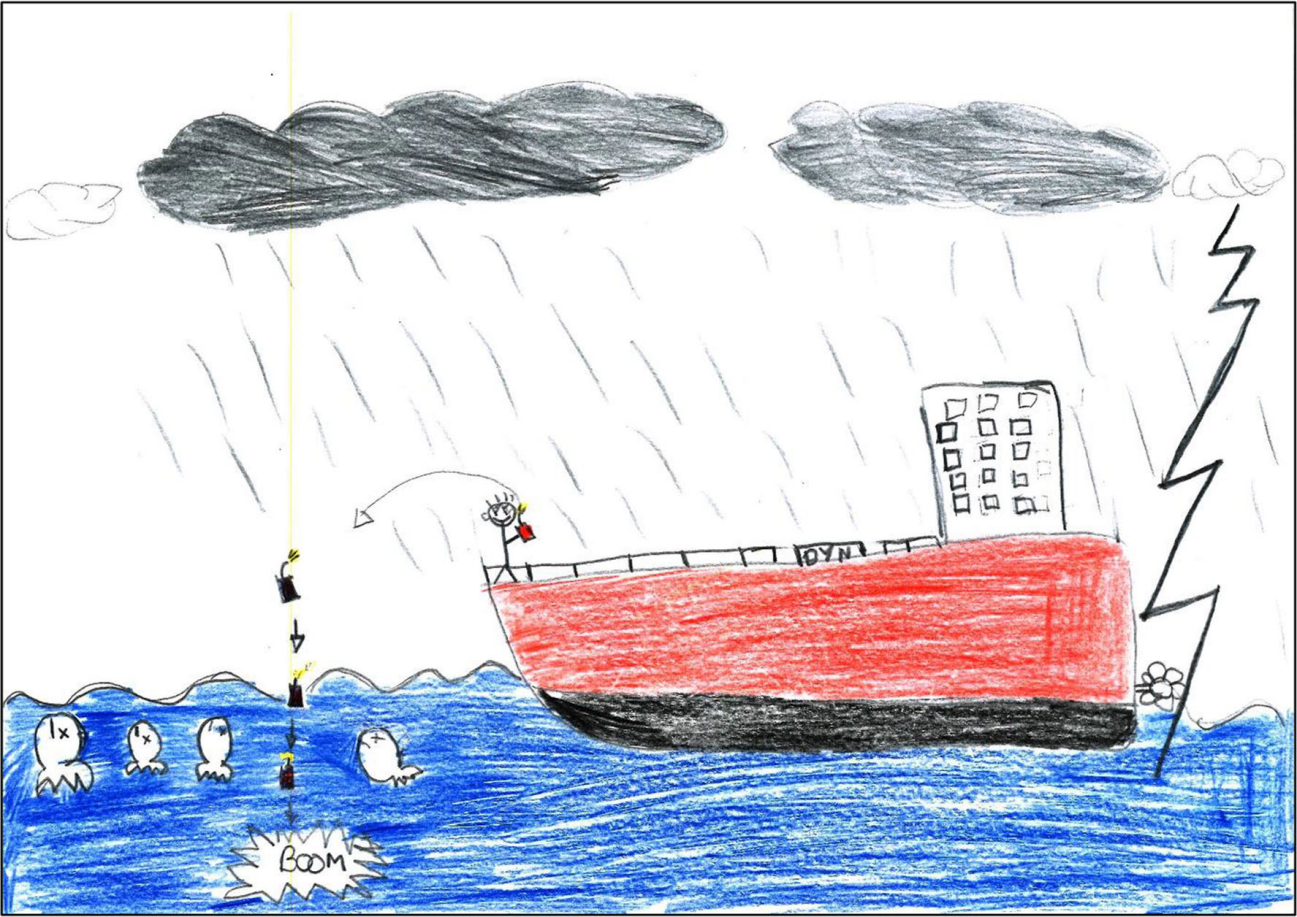
*“La pêche à la dynamite c’est pas bien”*—“Dynamite fishing is not good” (drawing made by a 10-year-old boy in New Caledonia’s rural site in November 2019)

Some drawings highlighted that land-sea territories are threatened by unsustainable fishing practices (that children had heard about, but not necessarily witnessed), with sometimes explicit references to management measures aiming to mitigate such practices, including surveillance and sanction. For instance, three pictures made by 12-year old children from the same class in Fiji’s urban site referred—in their own words—to “gillnet fishing”^5^ (Fig. 3). One of these children described such fishing in terms of “human activities that destroy sea creatures” (Fig. 3A); another one gave the following title to his drawing: “Don’t use gillnet fishing”, and explained that the helicopter he depicted is transporting “surveyors” (Fig. 3B); and a third one reckoned that fishing boats such as the one drawn catch too many fish and make too much money, while another watercraft was described as a “police boat” (Fig. 3C). These three drawings reflected the teacher’s efforts to raise her students’ awareness of various environmental issues, notably—shortly before our drawing workshop—of the negative impacts and necessary management of “gillnet fishing”. Another example: a drawing entitled *La pêche à la dynamite c’est pas bien* (“Dynamite fishing is not good”; Fig. 4), made by a 10-year old boy in New Caledonia’s rural site, represented a large vessel from which somebody is throwing dynamite in the sea. The detonation is signified by the onomatopoeia “boom”, and the fish thus rise to the surface, all dead as indicated by the cross in place of their eyes. The clouds are black, the rain is pouring, and a lightning strikes the water near the boat since, as the child explained, this fishing trip happens at night so that it is not spotted by the Navy, who could arrest, fine, and jail the offenders. In the interview, the child noted that this vessel is a *paquebot* (cruise ship), even though dynamite fishing—which he had never witnessed—is not practiced from this type of vessels. He also clarified that he had not heard about dynamite fishing at school, but from his father who used to fish this way, as well as via books and the internet.

Aside from these particularly striking drawings, during the interviews, some children made clear references to observations of, or concerns for, fishing of undersized or immature fish and overexploitation. In Fiji for example, an 11-year-old girl in the urban site said that she felt bad because *kawakawa* (groupers) are caught when they are too small, and a 12-year-old boy in the rural site mentioned that he told his parents they should protect small fish for the next generations. In New Caledonia, a 9-year old boy in the urban site represented a dolphin swimming away because the vessel nearby might catch it, and a 10-year-old girl in the rural site pointed out that, because people fish too much, there are not many fish left in the sea. Moreover, some children appeared concerned about the impacts on marine life of pollution from at-sea activities; a recurrent topic in the drawings, especially in Fiji’s urban site. For example, in a drawing entitled “Destroying sea life” (Fig. S10), a character is dumping drums of fuel from a ship into the sea.

The study therefore shows that, in both Fiji and New Caledonia, (at least some) children seem to be aware of two seemingly opposite, but interrelated dimensions of the ‘rush for Oceania’ (Allen et al. 2018), namely: exploitation and conservation of marine life (Fache et al. 2021). This suggests that children are clearly influenced by the environmental awareness-raising campaigns that are led by governmental agencies and/or non-governmental organizations (NGOs), and that contribute to reshape local relations with land-sea territories. For instance, children’s concerns in Fiji about fishing *kawakawa* and undersized or immature fish reflect two awareness-raising campaigns led by the Australian NGO cChange, involving a broad coalition of partners, based on a nation-wide, powerful communication strategy, using a variety of media (e.g., posters and stickers, radio, television, Facebook, YouTube), public events (e.g., on markets, at festivals, in schools), and role models like members of the national rugby team (Riera 2022). The first campaign, 4FJ (pronounce “for Fiji”), was initiated in 2014 to promote a seasonal fishing ban on grouper (*kawakawa*) and coral trout (*donu*) species, which was then formalized by the Ministry of Fisheries through the amendment of the *Offshore Fisheries Management Regulations 2014* with effect from 1 June 2019. The second, Set Size, was launched in 2017 by the Ministry of Fisheries to reverse the decline of inshore fisheries by urging fishers and consumers to avoid or release undersized fish. Beyond the influence of such campaigns, the interviews revealed that the children got information about the unsustainable fishing and marine pollution issues they highlighted from various sources, including their relatives, teachers (as part of the school curriculum, e.g., in biology and geography), books, television, and the internet.

However, although MPAs have become a cornerstone of marine conservation at the global level, the children’s drawings from both Fiji and New Caledonia did not refer to them, and they were mentioned (very briefly) in only four interviews. In particular, the Naiqoro Reserve in Fiji and the ZCNE in New Caledonia were not spontaneously brought to the fore by the children, neither in the drawings nor in the interviews, and our analysis has not revealed any influence of these spatial management measures on what the children chose to depict. This might partly be explained by the recentness of the establishment of the Naiqoro Reserve, and by some limitations in the efforts to raise awareness about the ZCNE among the local population. Clearly, our results might have been different if the drawing instruction given to the children had not focused on sea-related activities (“what you and others do in the sea”): the absence of these spatial management measures in the drawings and interviews could indeed be linked to the fact that, by definition, in a MPA some activities—particularly fishing—are prohibited.

### The sea as a ‘place-full’ space connecting human and more-than-human realms

Although many drawings show what might appear as simple and non-specific representations of fish, their drawers were able to identify the respective ethno-species^6^ in interviews. In those conducted in Fiji, the children mentioned in total more than 50 different ethno-species targeted or caught as part of the small-scale fisheries they pictured (Table S2). The five ethno-species they most frequently mentioned were *kawakawa* (grouper), *ta* (unicornfish), *kabatia* (emperor), *saqa* (trevally), and *qio* (shark). Two of these groups of fish, *kawakawa* and *kabatia*, are those women fishers mainly target for both food and income, with some species in rapid decline (Thomas et al. 2021). In the interviews conducted in New Caledonia, the children mentioned about 20 ethno-species (Table S3), in particular *dawa* (unicornfish), *rouget* (red snapper), *carangue* (trevally), *perroquet* (parrotfish), and *picot* (rabbitfish). The latter two are associated with spearfishing and angling, respectively, and were also among the most significant marine animals to local people in a previous survey (Sabinot et al. 2021).

**Fig. 5:**
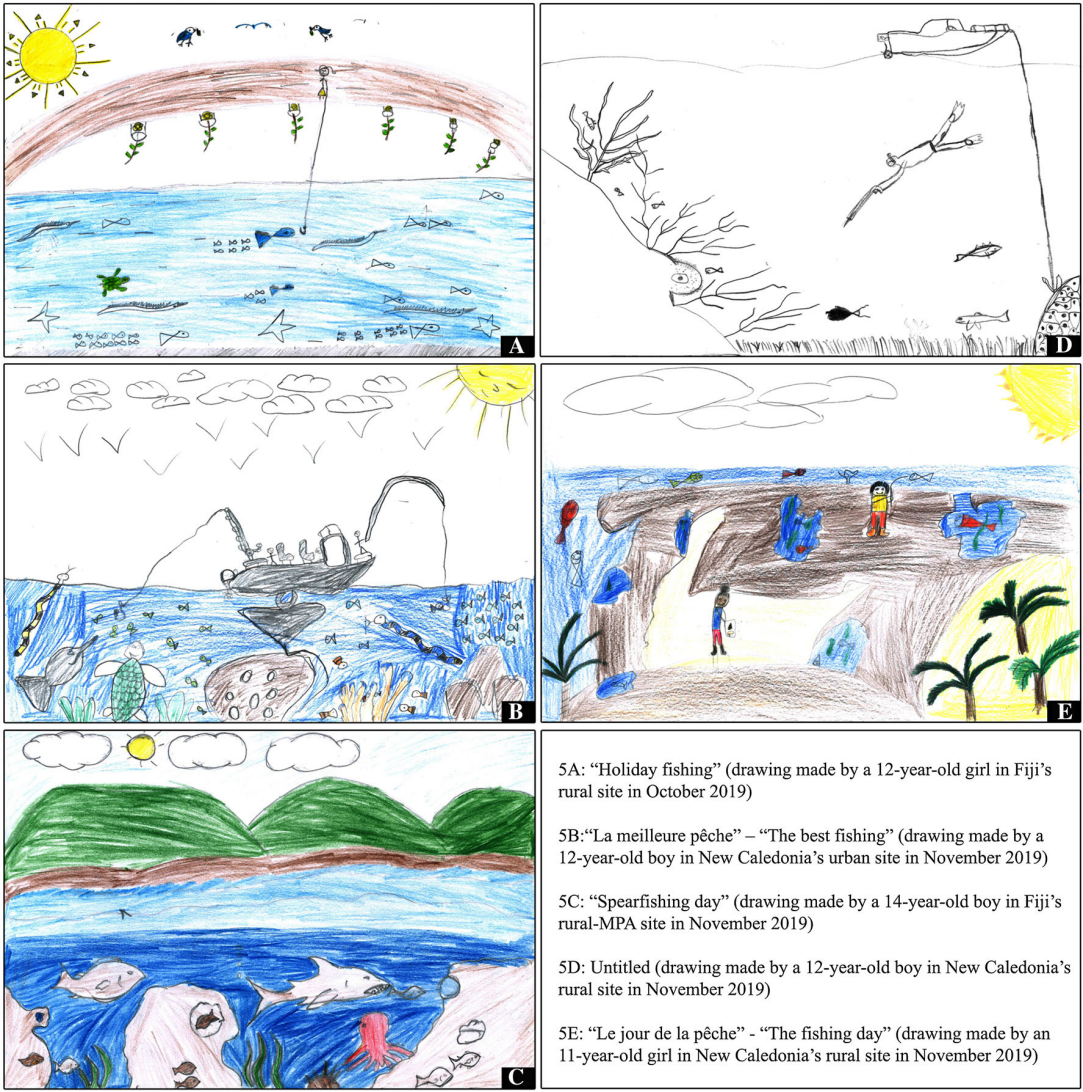
Five examples of representations of connections within/between species and/or habitats

In addition to fish that have a local value as food and/or source of income (see also Harding et al. 2022), the children’s drawings also included marine animals that are neither eaten nor sold, such as sea star or dolphin. They also highlighted marine animals, such as sea turtles and sharks, which are of both great ecological and cultural significance, and can therefore be considered as both ecological and cultural keystone species (Kitolelei et al. 2021). In Fiji, hawksbill (*Eretmochelys imbricata*) and green (*Chelonia mydas*) turtles hatch from nests in sandy beaches (Prakash et al. 2020), while the juveniles forage on the coral reefs and seagrass meadows along the coasts (Piovano et al. 2020). Among *iTaukei* people, “sea turtles are a form of spiritual and social property” (Morgan 2007, abstract), and presenting them to the paramount chief is part of the cultural obligations of the customary fishing clans (Kitolelei et al. 2022). The loggerhead (*Caretta caretta*) and the green turtles also nest on New Caledonian beaches and islets. The green turtle is called the “true turtle” in several *Kanak* languages because of its crucial customary significance and role (Sabinot and Bernard 2016). Sharks are present in 66 drawings, i.e., in 23% of all drawings (e.g., Figs. 2, 5C and S10), with some rare references to a risk of shark attack in New Caledonia (for topical reason and extensive media coverage at the time). Viviparous sharks such as the hammerheads use the sheltered shallow coastal waters as nurseries where neonates and young-of-the-year congregates (Marie et al. 2017). In Fiji, sharks are commonly caught by artisanal coastal fishers and have an increasing importance as a source of proteins (Glaus et al. 2019). In some parts of the country, “sharks are revered by the [*iTaukei*] people, who in turn are protected by them while at sea” (Veitayaki 2008, p. 119), as illustrated in our rural-MPA site where a “mutual respect between Kadavu people and sharks” has been documented (Gordon 2013, p. 208). New Caledonia hosts nearly 50 different species of sharks, all protected. It is rare not to encounter sharks when snorkelling or diving. Sharks are also very important for *Kanak* people, who consider them as grandfathers or ancestors and owe them great respect (e.g., Leblic 2008).

Whatever their fishing experience, many children drew the marine underwater world, including marine animals and plants therein (Fig. S11). In the interviews, they pointed out the interrelatedness among these entities or between the latter and their habitats. Some drawings referred to relational behaviors within a species, for instance juveniles and adults hanging out together (e.g., Fig. 5A where four “mother” fishes are followed by their “babies”), or schooling (e.g., Fig. 5B where the fish—that the child named *relégués*, *Terapon jarbua*—are swimming together in the same direction). Other drawings showed marine animals in their ecosystems, i.e., with other species and/or within a specific habitat. In the bottom centre of Fig. 5B for example, one can see—as explained by the child—clownfish in and around sea anemones, as well as shellfish on a coral potato. In the bottom centre of Fig. 5C, a fish is entering a hole in the brown shape, which the child identified as coral, while a shark is chasing another fish, which refers to a food chain step. In Fig. 5D, marine animals and plants are depicted in the outer slope of a reef, while Fig. 5E represents the reef flat at low tide, with only “small pools” remaining, where one can find seaweeds, shellfish, and fish.

This demonstrates that some children of our target age group have already acquired a high level of ecological knowledge, which is revealed by their accurate representation of complex ecosystems, including interactions within and/or between species. For (at least some) children in Fiji and New Caledonia, fishing—as a mode of “active engagement with the constituents of [their] surroundings” (Ingold 2000, p. 5), namely of land-sea territories—represents a way of knowing marine environments and their various dwellers. In particular, these children often go fishing with relatives or friends, which allows for intra- and/or inter-generational learning, through horizontal and/or vertical mechanisms of knowledge transmission (Porcher et al. 2022). Despite observations that IFK is increasingly threatened in the Pacific (Kitolelei et al. 2021), our study shows that its transmission through hands-on experiences is still occurring, and that children’s interest in learning IFK is palpable.

### Research perspectives and policy recommendations

The limitations of our use of children’s drawings as a research tool included the following: the drawing instruction, consisting of a single and overarching question, circumscribed the information that was elicited; the drawings were highly influenced by the spatial–temporal and interactional context in which they were produced; and the shortness of the interviews prevented us from delving deeper into children’s perceptions on marine sustainability (Fache et al. 2022). In the future, these limitations could be overcome and our findings cross-checked through a second series of drawing workshops, based on three drawings relating to the three themes highlighted in this study, with more in-depth interviews. Future research could also use other methodologies to tackle the new or reformulated research questions that emerged from the study, including the extent to which children (1) interpret boats, turtles, and waste (among other entities) as epitomizing ridge-to-reef-to-ocean connectivity, (2) increasingly conform to a gendered division of labor in small-scale fisheries as they age, and (3) are not only recipients but also producers of IFK. These methodologies could either be those conventional in social sciences, such as a combination of participant observation and semi-structured interviews, or be based on visual approaches that facilitate the co-production of knowledge, such as photo-ethnography (Gearhart 2013; Jankowski et al. 2015) or stop-motion storytelling (Gorman et al. 2022).

Overall, the study has highlighted children’s views that could be incorporated into a child rights and intergenerational justice approach to marine sustainability, aiming at policies that are made in the best interests of, and make sense for, those whose future is most at stake. First, children’s representations of the sea as inextricably tied to the land call for strengthening regional^7^ and national ridge-to-reef programs, while/by facilitating children’s participation in their scaling-up. Second, children’s awareness of the exploitation-conservation nexus calls for a systematic assessment of the influence of awareness-raising campaigns on the young public, while ensuring that these are based on (rather than ignore) children’s IFK—a double objective to which children’s drawings can contribute (e.g., Chabanet et al. 2018). Third, children’s insight into the need to protect ecological-and-cultural keystone species for future generations calls for an increased focus of conservation efforts and management strategies on such species, which could result in an increased effectiveness in maintaining or restoring both ecosystem and human health, while acknowledging the values, practices, rights and interests of their customary stewards, including children and future generations (Garibaldi and Turner 2004; Noble et al. 2016).

## Conclusion

Based on children’s drawings and their own comments on the latter, this study highlights that children from Fiji and New Caledonia see the Pacific Ocean as a web of ecological connections (e.g., between the sea and the land, within/between marine species, between species and habitats), as well as sociocultural connections (e.g., place attachment, cultural keystone species, IFK transmission). This study also spotlights that small-scale fisheries are an essential way of connecting people (especially children), the sea, and all living things therein, and of maintaining these connections over time, despite environmental issues that are both local and global in scope. Finally, we illustrate that drawings are a relevant interdisciplinary tool to explore and make accessible children’s perceptions on sustainable futures, in Oceania and beyond.

## Supplementary Information

Below is the link to the electronic supplementary material.

Supplementary file1 (PDF 2122 KB)

## Footnotes

1 See the full version of UNCRC’s Article 12 here: https://www.unicef.org.uk/wp-content/uploads/2010/05/UNCRC_PRESS200910web.pdf; its summary version here: https://www.unicef.org.uk/wp-content/uploads/2019/10/UNCRC_summary-1_1.pdf; and its simplified version here: https://www.cypcs.org.uk/rights/uncrc/articles/article-12/.

2 *Fisheries (Naiqoro Passage Spawning Aggregation Marine Reserve), Sect. 9 of the Fisheries Act 1941, Legal Notice N°4*, https://www.fiji.gov.fj/getattachment/b920c7d1-60e8-4b81-aae8-343b3d2c3730/LN-4---Fisheries-(Naiqoro-Passage-Spawning-Aggrega.aspx (Accessed on 26 July 2021).

3 https://whc.unesco.org/en/list/1115/ (Accessed on 26 July 2021).

4 Translated into *iTaukei* language in Fiji and French in New Caledonia.

5 Even though the nets these children drew look like purse seine nets rather than gillnets.

6 Vernacular species, which can match or differ from scientific species, for instance with different local names given to the same scientific species depending on its life stage, or conversely, a single local name given to different scientific species.

7 https://www.pacific-r2r.org/how-we-operate (Accessed on 3 May 2022).
