# Supplementary material for: “Draw the sea…”: Children’s representations of ocean connectivity in Fiji and New Caledonia
Source: Ambio. 2022 Sep 23;51(12):2445–58. doi: 10.1007/s13280-022-01777-1 (PMC9584002; doi:10.1007/s13280-022-01777-1)
Supplement: Supplementary file 1 — Supplementary file1 (PDF 2122 KB) [file 13280_2022_1777_MOESM1_ESM.pdf]

Ambio

Electronic Supplementary Material
This supplementary material has not been peer reviewed.

Title: **"Draw the sea…": Children's representations of ocean connectivity in Fiji and New Caledonia**

Authors: Elodie Fache, Susanna Piovano, Alisi Soderberg, Malakai Tuiono, Léa Riera, Gilbert David, Matthias Kowasch, Simonne Pauwels, Annette Breckwoldt, Stéphanie M. Carrière, Catherine Sabinot

Figure S1: Quantitative assessment of the presence of a land-sea continuum in children's drawings.

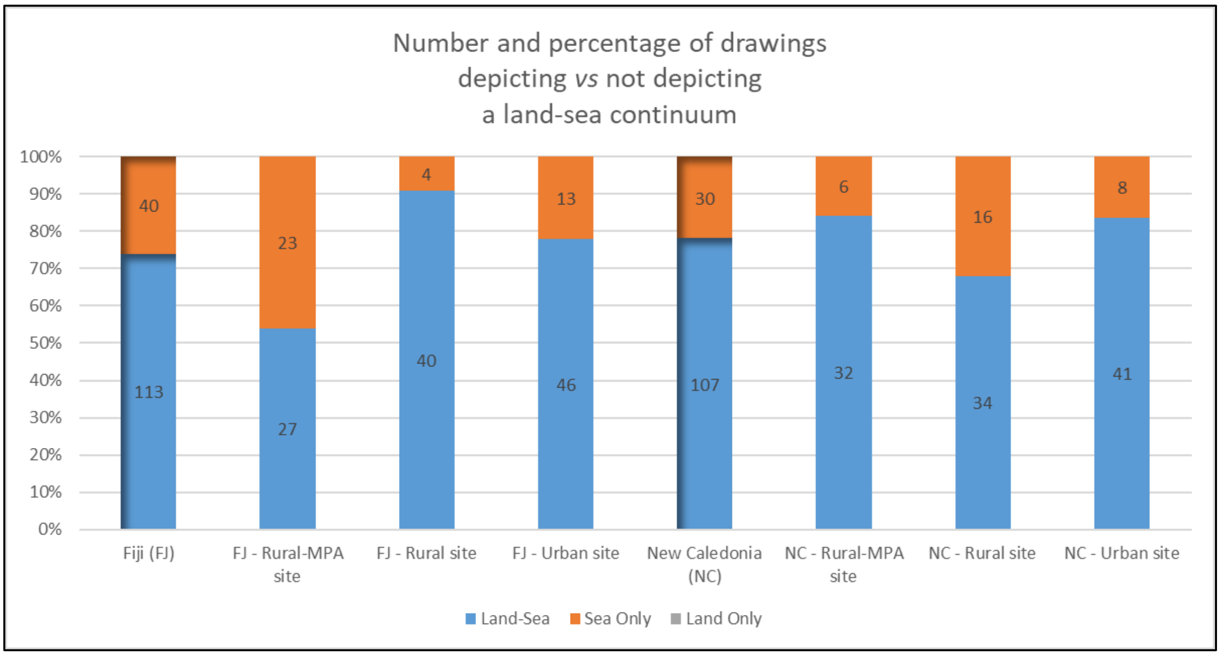

Figure S2: Examples of drawings including small-motorized boats.

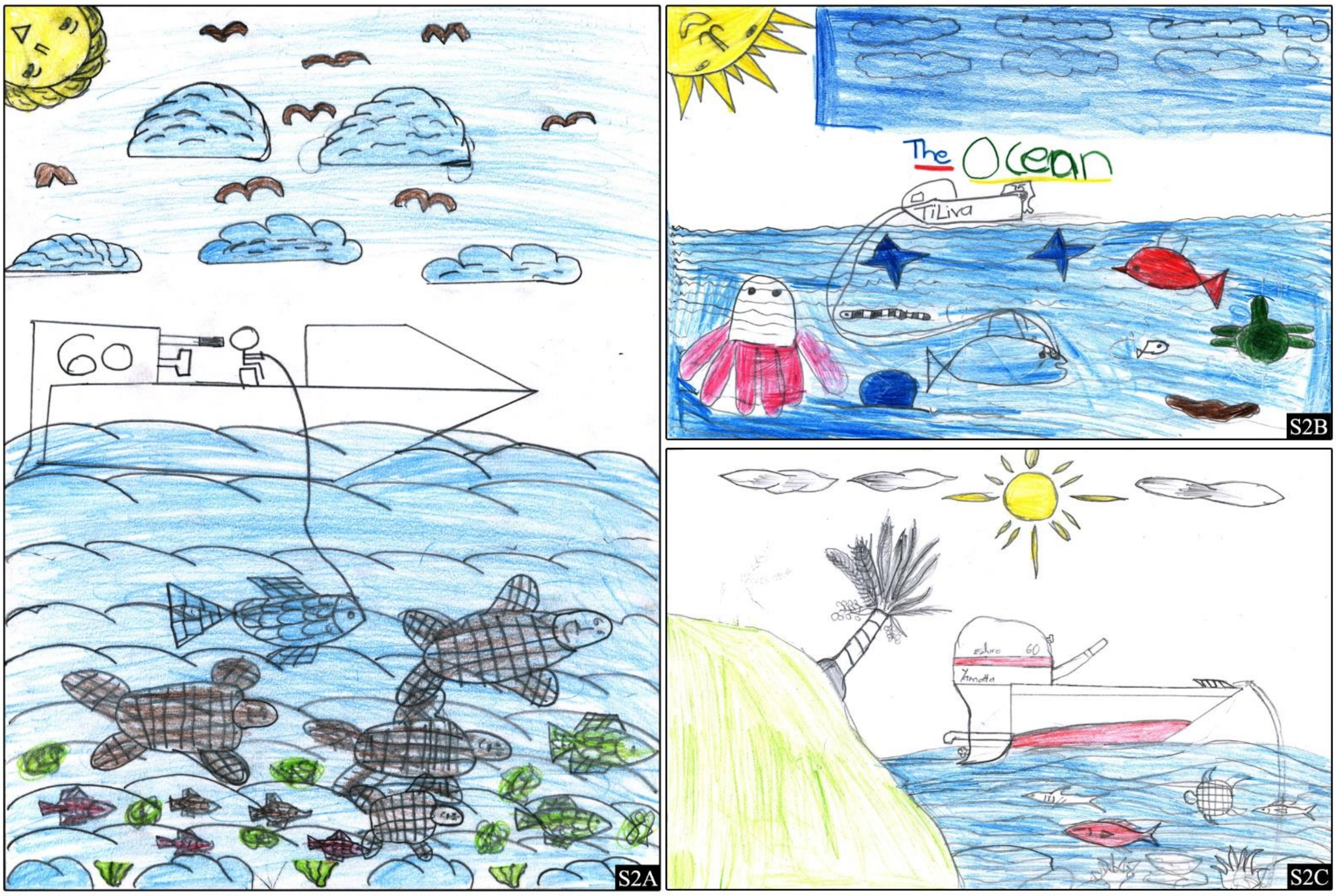

S2A: “Different kinds of fish” (drawing made by a 12-year-old girl in Fiji’s rural-MPA site in November 2019)

S2B: “The living animals in the sea” (drawing made by a 12-year-old girl in Fiji’s rural-MPA site in November 2019)

S2C: “The ocean” (drawing made by an 11-year-old boy in Fiji’s rural-MPA site in November 2019)

Figure S3: *“La merveilleuse plage”* - “The wonderful beach”
(drawing made by a 10-year-old girl in New Caledonia’s rural site in November 2019).

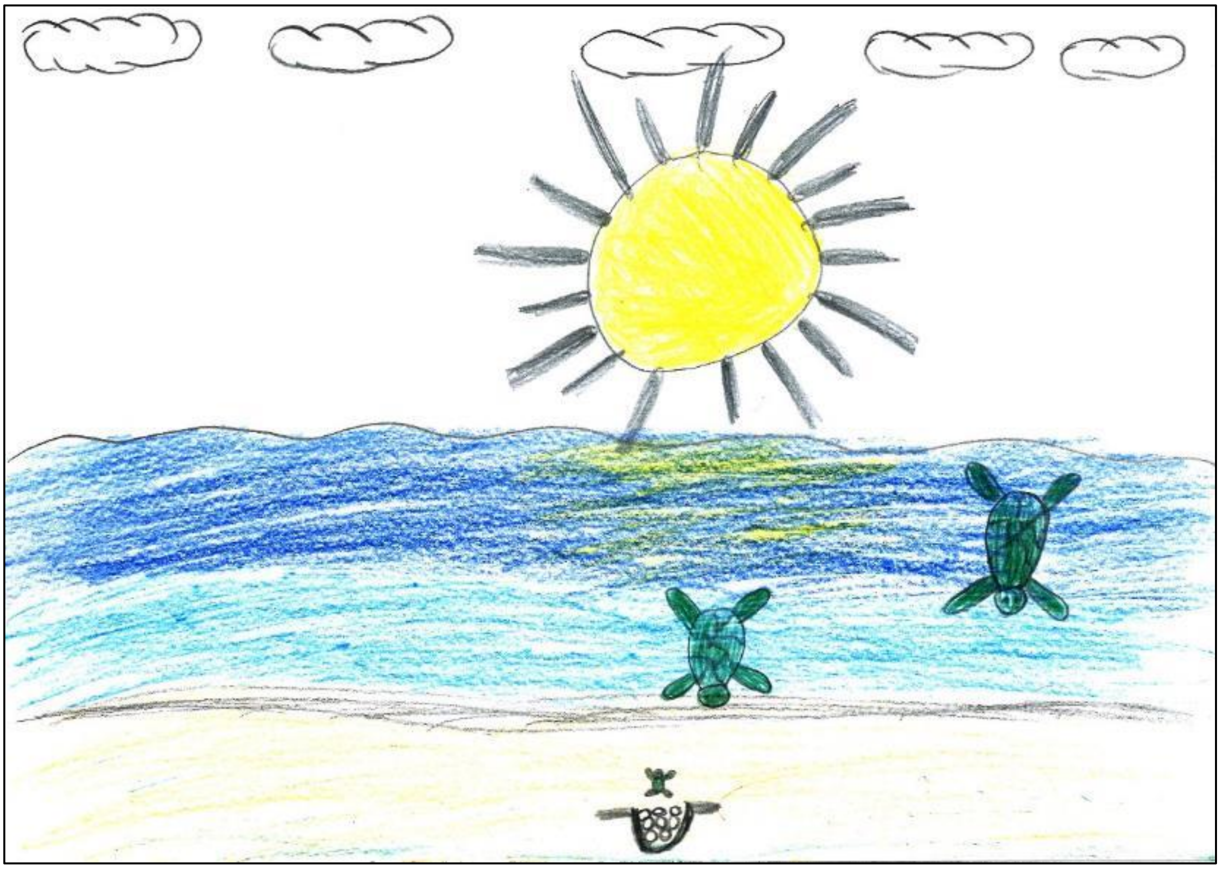

Figure S4: Examples of drawings highlighting waste management issues.

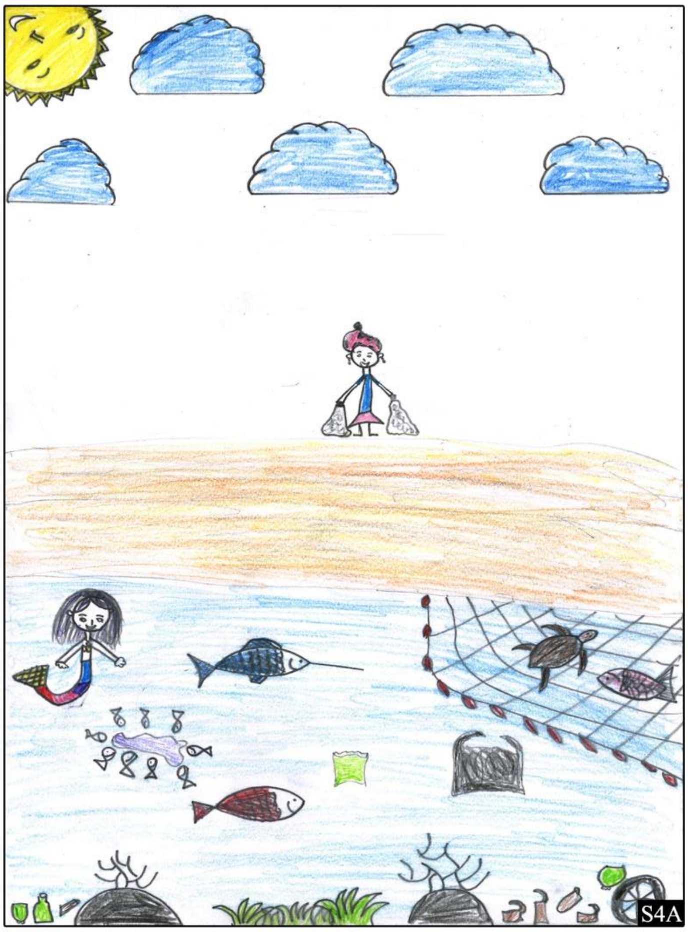

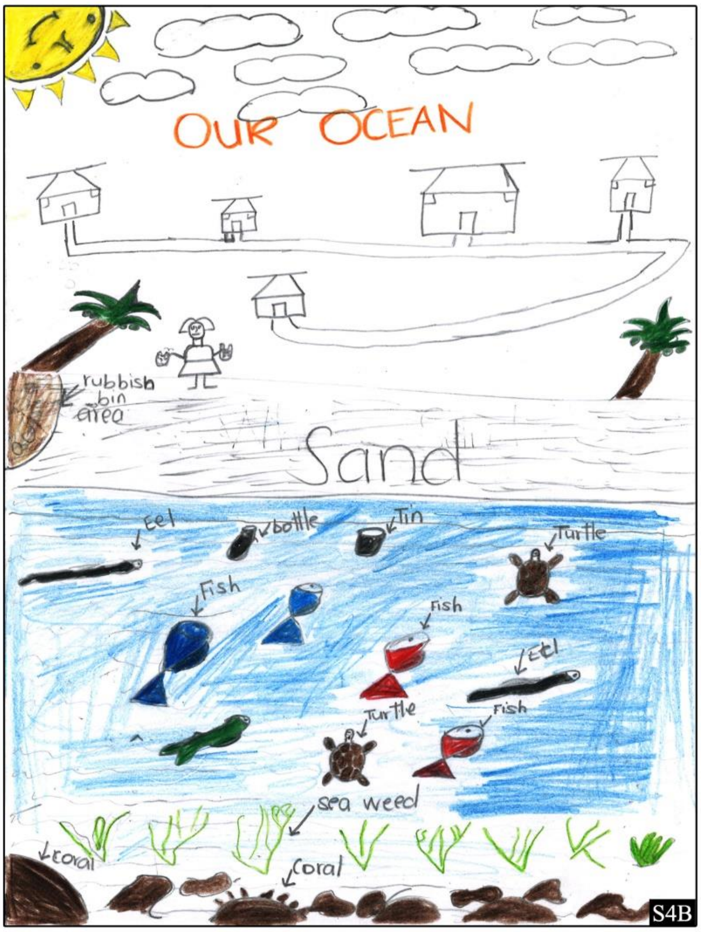

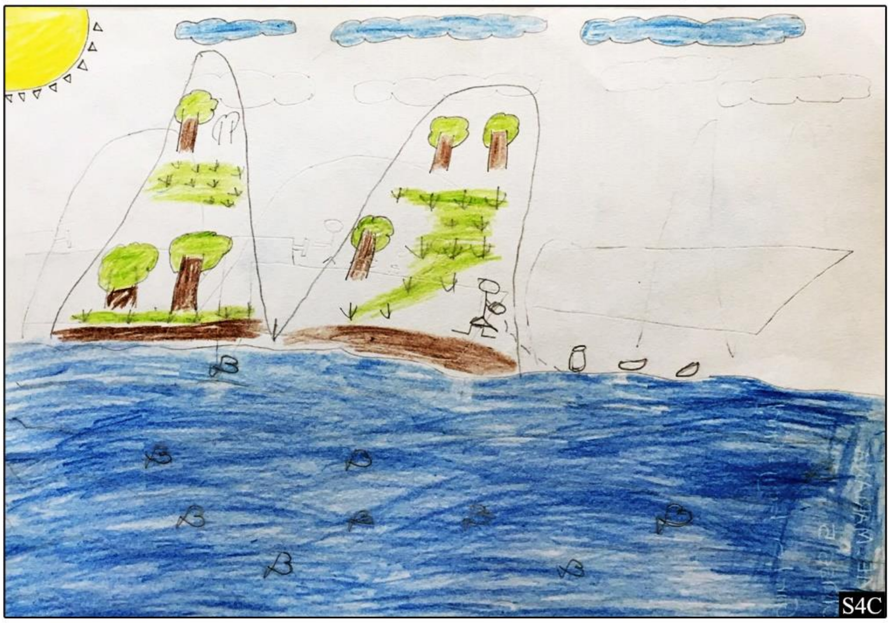

S4A: “Stop polluting the sea” (drawing made by a 10-year-old girl in Fiji’s rural-MPA site in November 2019)

S4B: “Sea pollution” (drawing made by a 14-year-old girl in Fiji’s rural-MPA site in November 2019)

S4C: “Don’t put rubbish carelessly” (drawing made by an 11-year-old girl in Fiji’s rural-MPA site in November 2019)

Figure S5: Quantitative assessment of the types of place represented in children's drawings.

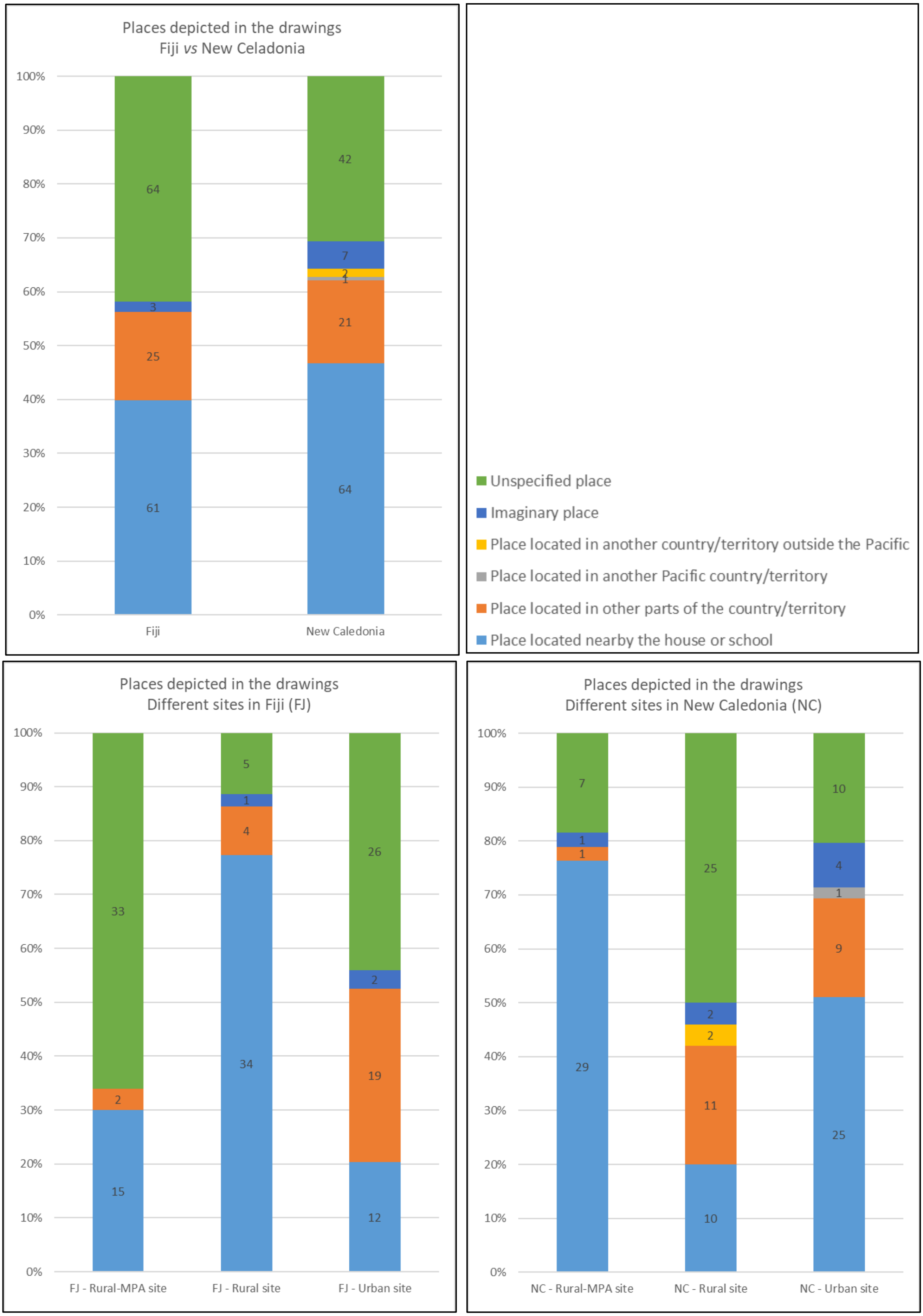

Figure S6: Quantitative assessment of the presence of fishing activities in children's drawings.

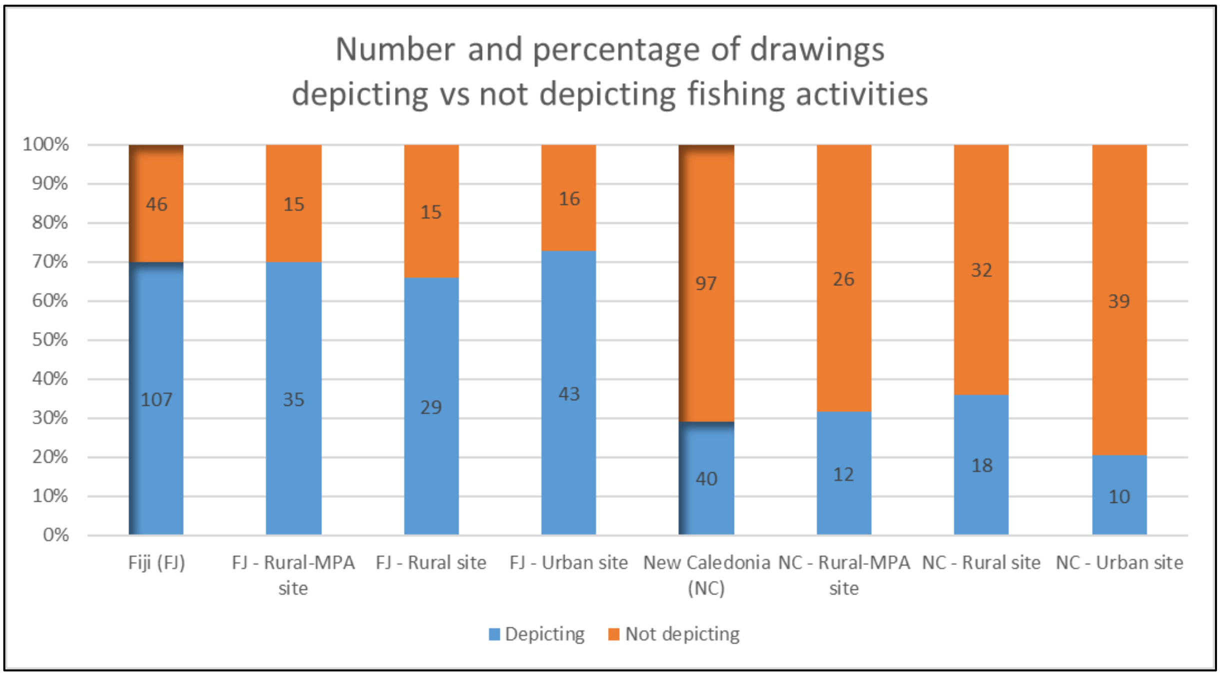

Figure S7: Influence of gender on the representation of fishing activities.

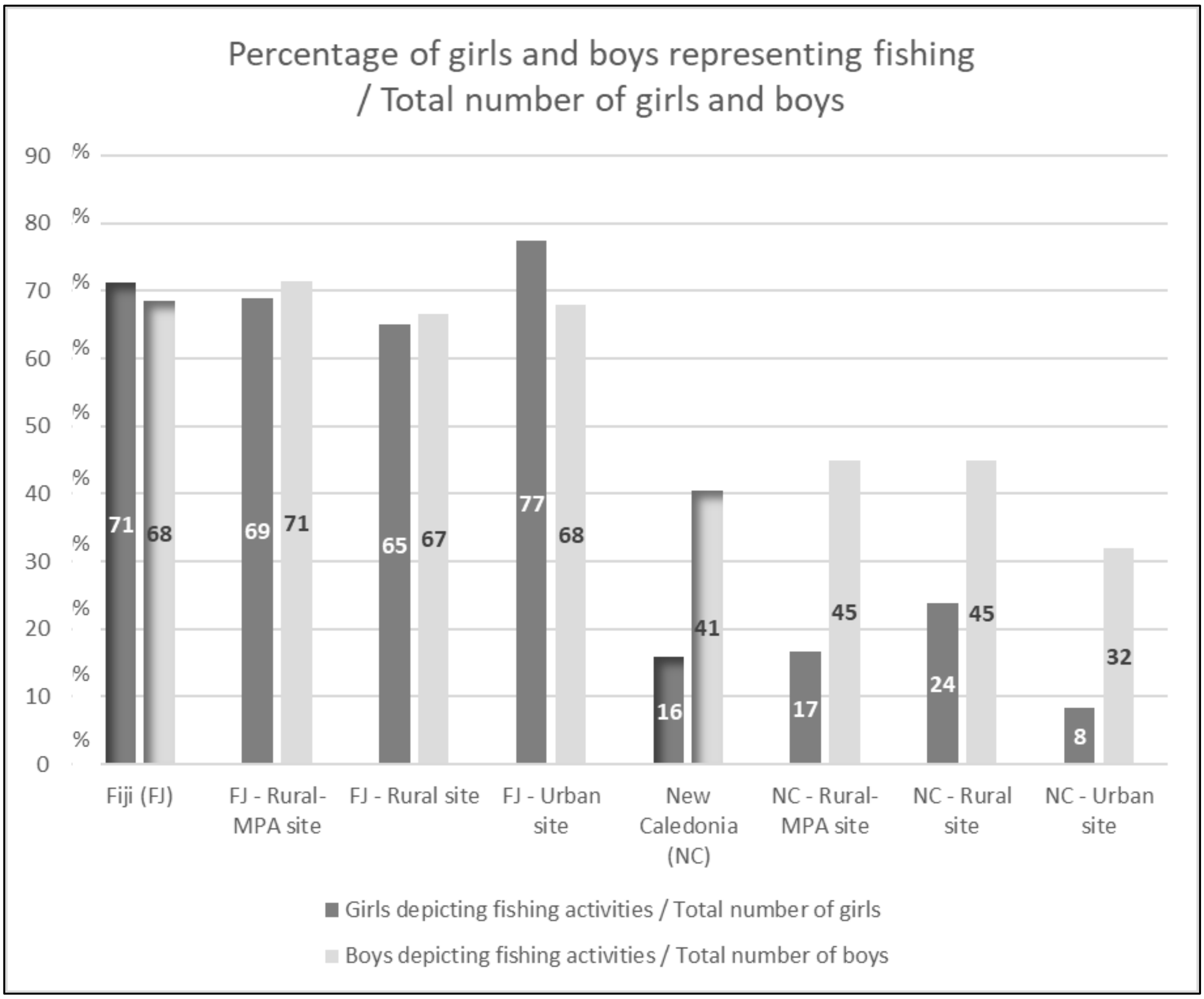

Figure S8: Quantitative assessment of the gender of the fishers (other than themselves) drawn by girls (S8A) versus boys (S8B).

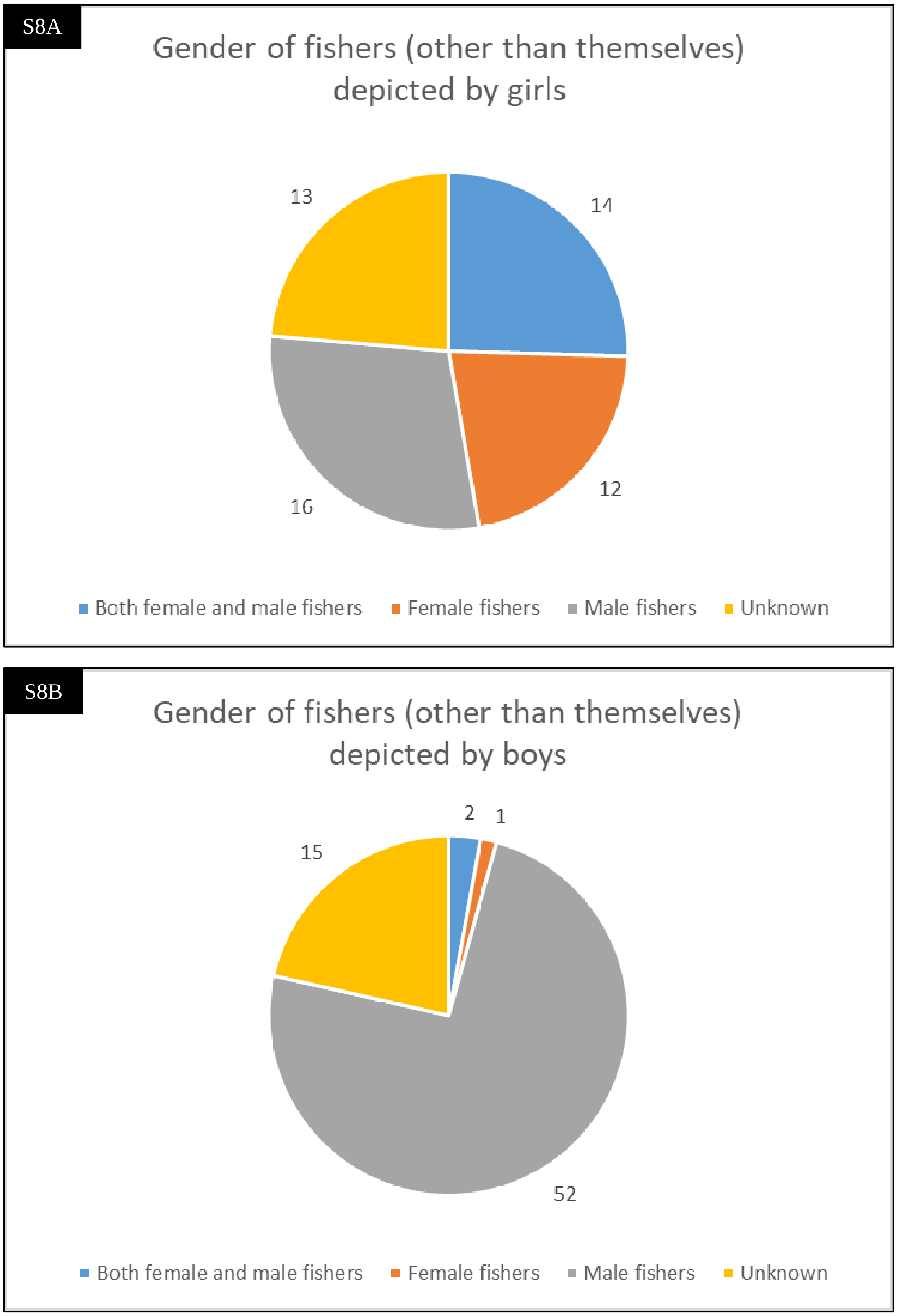

Figure S9: Five drawings reflecting a clear gender differentiation in fishing locations and techniques.

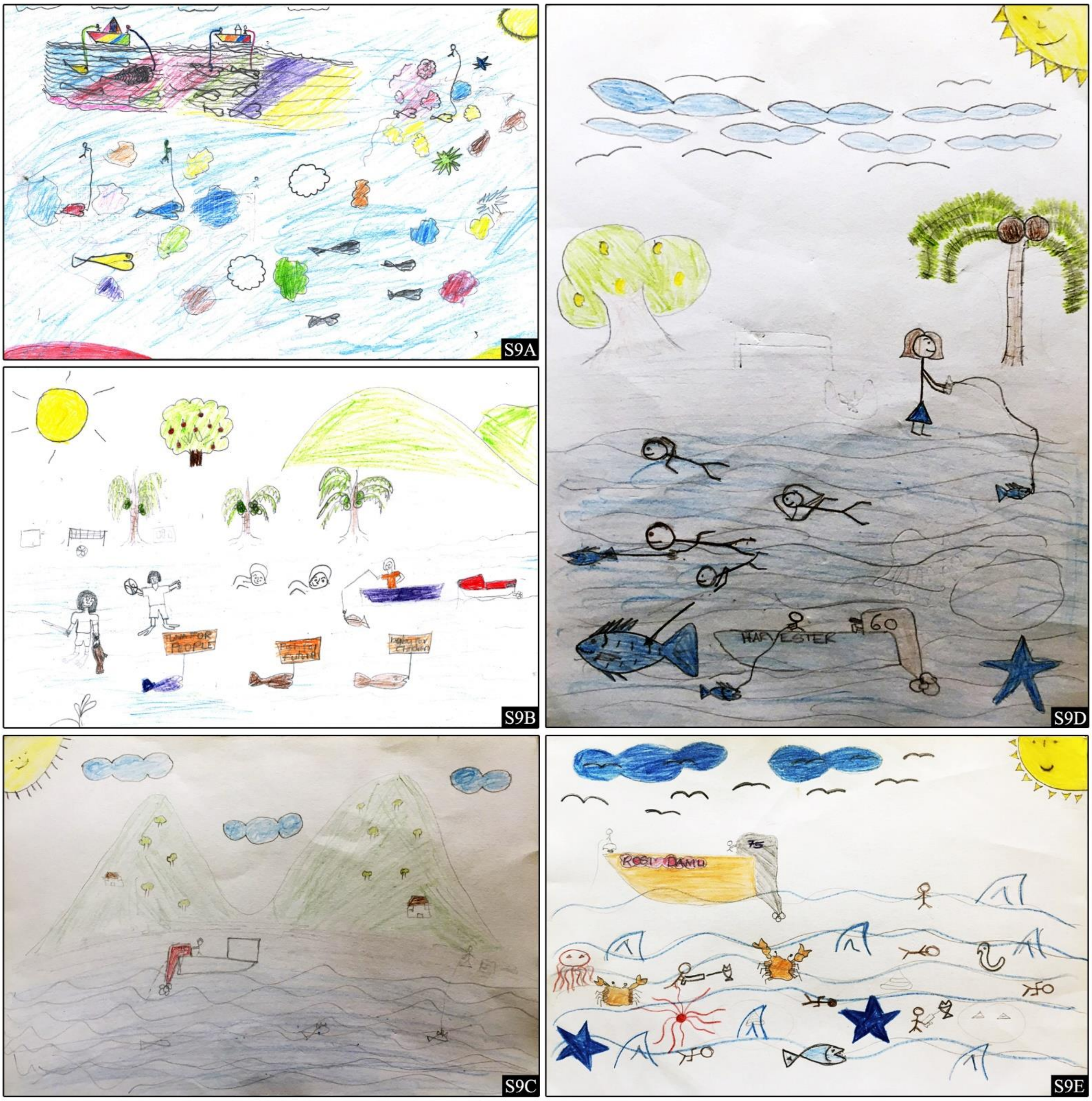

S9A: “Going fishing” (drawing made by an 11-year-old boy in Fiji’s rural site in September 2019)
In this drawing, boys are line-fishing from a watercraft in deeper water, while girls are line-fishing from the shore.

S9B: “Keeping marine sea” (drawing made by a 13-year-old girl in Fiji’s rural site in October 2019)
In this drawing, the child’s mother is line-fishing from a watercraft, while her father is fishing with a speargun.

S9C: “The things we get from the sea” (drawing made by a 10-year-old girl in Fiji’s rural-MPA site in November 2019)
In this drawing, the child’s mother is line-fishing from the shore, while her father is line-fishing from a watercraft.

S9D: Picnic (drawing made by a 9-year-old girl in Fiji’s rural-MPA site in November 2019)
In this drawing, the child’s mother is line-fishing from the shore, and her sister is line-fishing from a watercraft, while her father and brother are fishing with a hand-spear.

S9E: “Things to do to get money” (drawing made by a 12-year-old girl in Fiji’s rural-MPA site in November 2019)
In this drawing, the child and her girlfriends are line-fishing from a watercraft, while her boyfriends are fishing with a speargun.

Figure S10: “Destroying sea life”
(drawing made by an 11-year-old boy in Fiji’s urban site in September 2019).

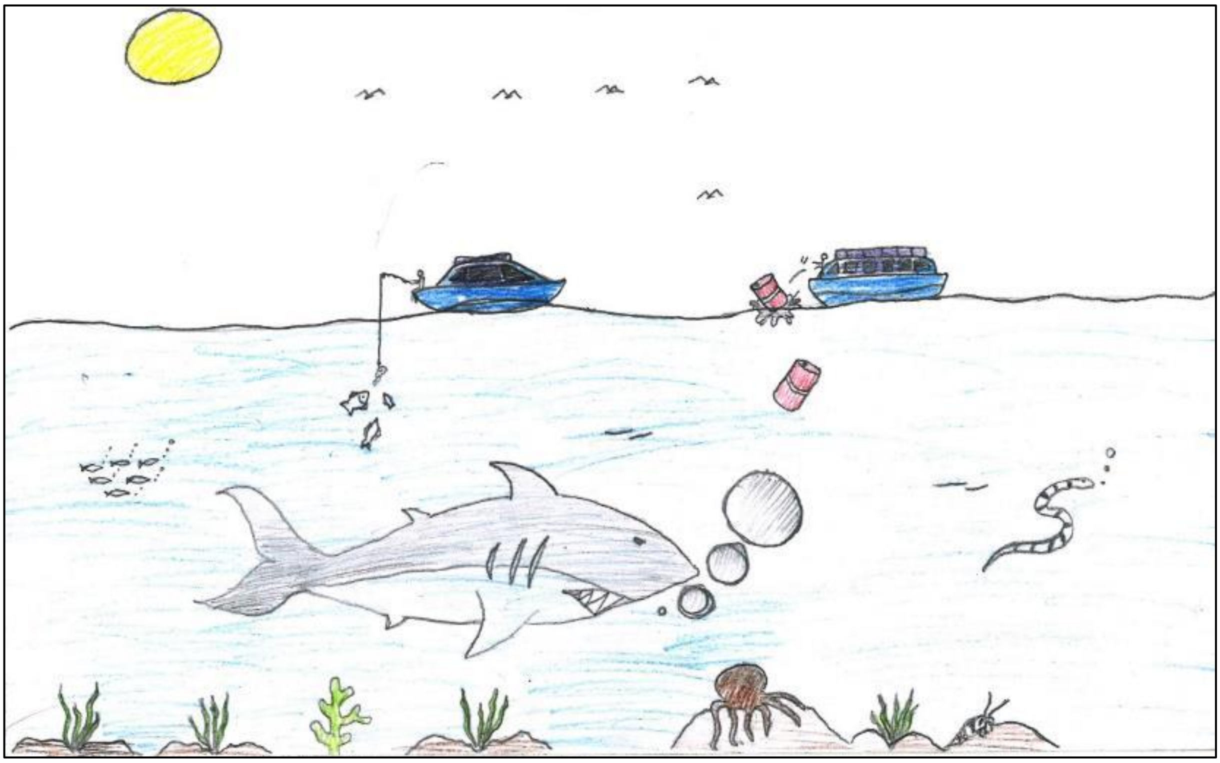

Figure S11: Quantitative assessment of the presence of an underwater view (S11A), marine animals other than corals (S11B), and corals, seagrasses or seaweeds (11C), in children's drawings.

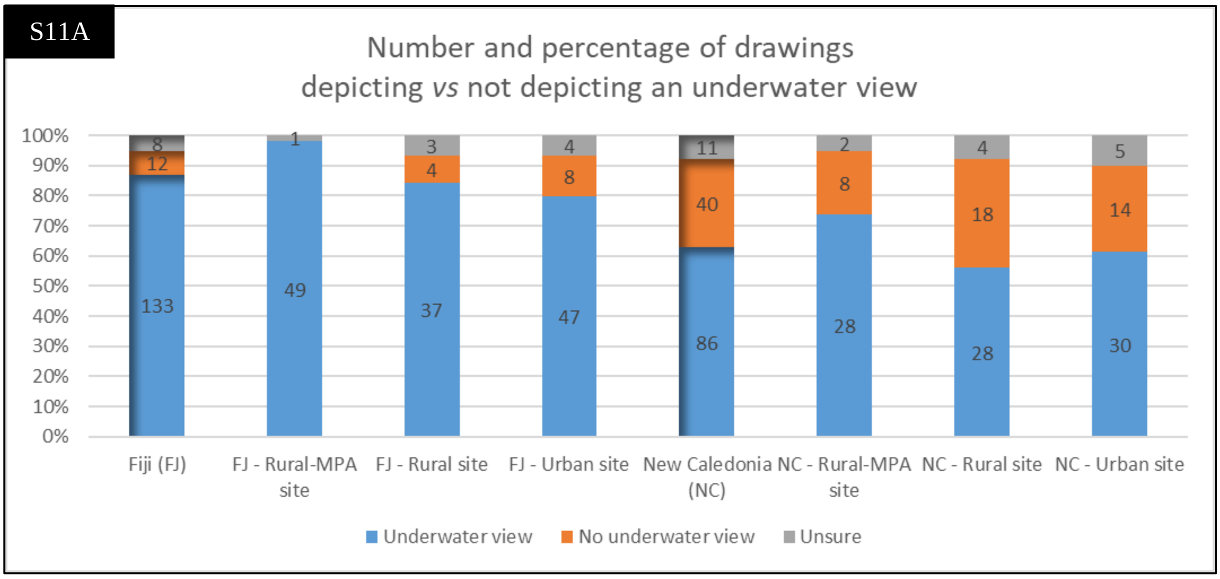

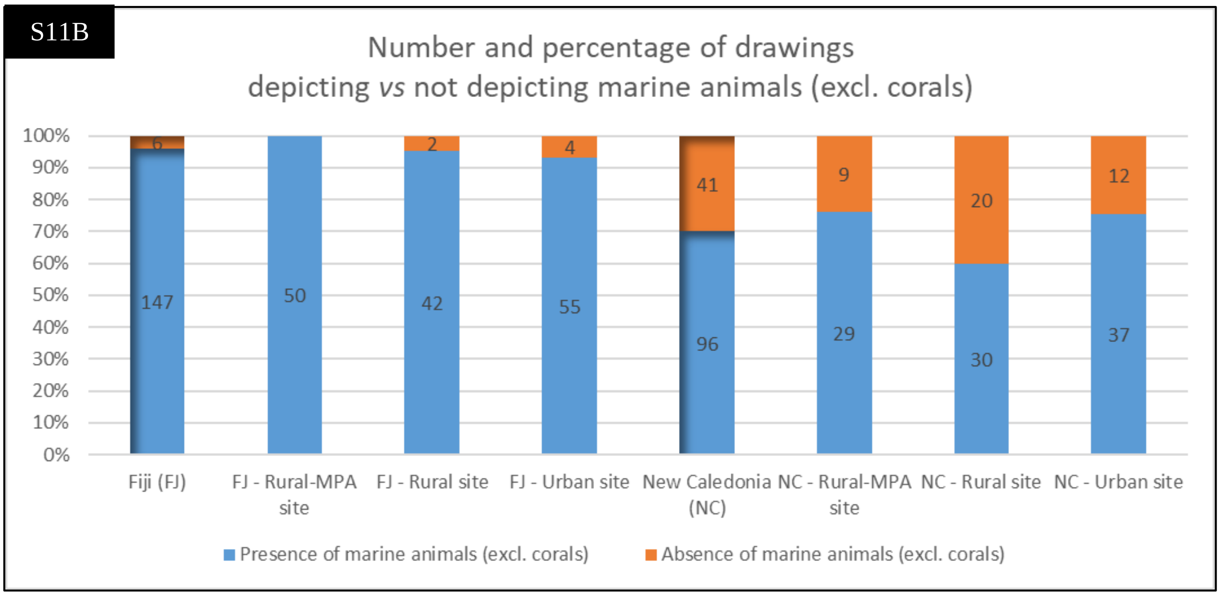

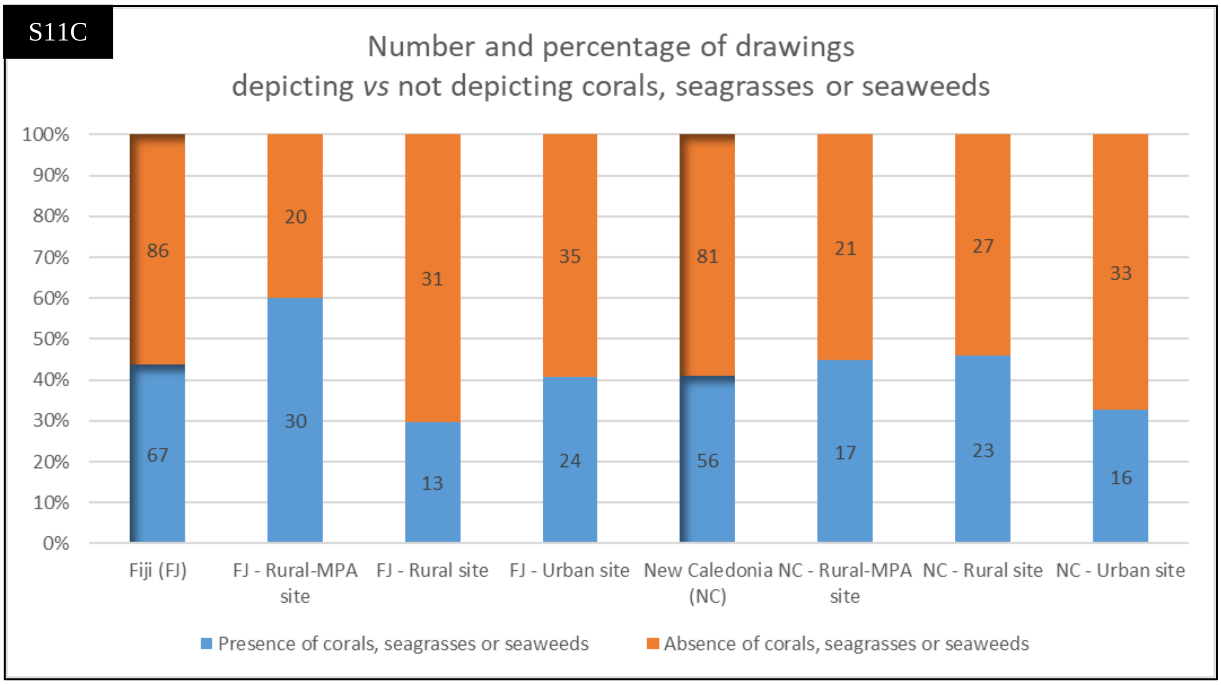

Table S1: Number of participants in each site (NC = New Caledonia).

	Fiji, Urban (Suva/Lami)	Fiji, Rural (Cicia)	Fiji, Rural with MPA (Kadavu)	NC, Urban (Nouméa)	NC, Rural (Yaté)	NC, Rural with MPA (Hienghène)
Number of participants	59	44	50	49	50	38

Table S2: List of 50 ethno-species mentioned by children in Fiji in interviews (by alphabetical order), based on the team's interview notes.

iTaukei name	English name	Group	Taxon
Balagi	Fine-lined surgeonfish	Fish	*Acanthurus grammoptilus*
Bati	Red bass	Fish	*Lutjanus bohar*
Bumarawa	Parrotfish	Fish	Family Scaridae
Cucu	Goatfish	Fish	*Parupeneus* spp.
Cumu	Triggerfish	Fish	Family Balistidae
Damu	Mangrove red snapper	Fish	*Lutjanus argentimaculatus*
Donu	Coral trout	Fish	*Plectropomus* spp.
Drabua	Sabre squirrelfish	Fish	*Sargocentron spiniferum*
Draunikura	Wrasse	Fish	*Cheilinus* spp.
Droudroua	Leopard coral grouper	Fish	*Plectropomus leopardus*
Dulu (same as Ogo)	Great barracuda	Fish	*Sphyraena barracuda*
Duna	Freshwater eel	Fish	*Anguilla marmorata*
Ikaloa (same as Kawakawa loa)	Peacock hind, bluespotted grouper, celestial grouper, roi	Fish	*Cephalopholis argus*
Jila	Lined surgeonfish	Fish	*Acanthurus lineatus*
Kabarara (same as Qitawa)	Crescent grunter	Fish	*Terapon jarbua*
Kabatia	Red-eared emperor	Fish	*Lethrinus rubrioperculatus*
Kai	Freshwater mussel	Mollusc	*Batissa violacea*
Kake	Snapper	Fish	*Lutjanus* spp.
Kanace	Mullet	Fish	Family Mugilidae
Karakarawa	Highfin parrotfish	Fish	*Scarus longipinnis*
Katavatu	Rugose giant clam	Mollusc	*Tridacna maxima*
Kawakawa loa	Peacock grouper	Fish	*Cephalopholis argus*
Kawakawa	Grouper	Fish	*Epinephelus* spp.
Kuita	Octopus	Mollusc	*Octopus cyanea*
Lokoloko ni qio/Kalokalo	Starfish	Sea star	*Linckia laevigata*
Misimisi	Wrasse	Fish	*Thalassoma* spp.
Nuqa	Rabbitfish	Fish	*Siganus* spp.
Ogo	Great barracuda	Fish	*Sphyraena barracuda*
Ose	Yellowfin goatfish	Fish	*Mulloidichthys vanicolensis*
Qio	Shark	Fish	Several species
Qitawa	Crescent grunter	Fish	*Terapon jarbua*
Saqa	Giant trevally	Fish	*Caranx ignobilis*
Senigaragara	Honeycomb grouper	Fish	*Epinephelus merra*

Sirisiriwai	Painted or silver sweetlip	Fish	*Diagramma pictum*
Sokisoki	Porcupinefish	Fish	*Diodon hystrix*
Ta qio	Bulbnose unicornfish	Fish	*Naso tonganus*
Ta	Bluespine unicornfish	Fish	*Naso unicornis*
Tabace	Whitespotted surgeonfish	Fish	*Acanthurus guttatus*
Tarase	Deep water redfish	Sea cucumber	*Actinopyga echinites*
Tarosese	Titan triggerfish	Fish	*Balistoides viridescens*
Tivitivi	Butterflyfish	Fish	Family Chaetodontidae
Tugadra	Bigeye scad	Fish	*Selar crumenophthalmus*
Tuna	Tuna	Fish	Family Scombridae
Ulavi	Parrotfish	Fish	Family Scaridae
Ulurua	Steephead parrotfish	Fish	*Chlorurus microrhinos*
Urau	Lobster	Crustacean	Family Palinuridae and Scyllaridae
Vai	Ray	Fish	Family Dasyatidae and Mobulidae
Vivili	Collective term used for shells	Mollusc	Several species
Vo	1. Dusky sleeper<br>2. Broadhead sleeper	Fish	*1. Eleotris fusca*<br>*2. E. melanosoma*
Vonu	Sea turtle	Reptile	Family Cheloniidae

Table S3: List of 20 ethno-species mentioned by children in New Caledonia in interviews (by alphabetical order), based on the team's interview notes.

Local name	English name	Group	Taxon
Araignée	Giant spider conch	Mollusc	*Lambis truncata*
Bec de canne	Spangled emperor	Fish	*Lethrinus nebulosus*
Bossu d'herbe	Pink ear emperor	Fish	*Lethrinus lentjan*
Carangue	Trevally	Fish	*Caranx* spp. / *Carangoides* spp.
Coquillages non spécifiés	Shellfish	Mollusc	Several species
Dawa	Bluespine unicornfish	Fish	*Naso unicornis*
Langouste	Lobster	Crustacean	*Panulirus* spp.
Mulet	Mullet	Fish	*Mugil* spp.
Perroquet	Parrotfish	Fish	Family Scaridae
Perroquet à bosse	Bumphead parrotfish	Fish	*Bolbometopon muricatum*
Picot	Rabbitfish	Fish	*Siganus* spp.
Porcelaine	Cowrie	Mollusc	*Cypraea* spp.
Relégué	Crescent grunter	Fish	*Terapon jarbua*
Rouget	Mangrove red snapper	Fish	*Lutjanus argentimaculatus*
Sardine	Sardines	Fish	Family Engraulidae
Saumonée	Leopard coral grouper	Fish	*Plectropomus leopardus*
Thazard	Narrow-barred spanish mackerel	Fish	*Scomberomorus commerson*
Tortue	Sea turtle	Reptile	Family Cheloniidae
Troca	Trochus	Mollusc	*Rochia nilotica*
Wiwa	Blue sea chub	Fish	*Kyphosus cinarescens*
